# An overview of co-morbidities and the development of pressure ulcers among older adults

**DOI:** 10.1186/s12877-018-0997-7

**Published:** 2018-12-11

**Authors:** Efraim Jaul, Jeremy Barron, Joshua P. Rosenzweig, Jacob Menczel

**Affiliations:** 1 Skilled Geriatric Nursing Department, Herzog Medical Center, 3900, 91035 Jerusalem, Israel; 2 Affiliated with the Hebrew University Medical School, Jerusalem, Israel; 3 Chronic Ventilation Department, Herzog Medical Center, Jerusalem, Israel; 4 Johns Hopkins University, Baltimore, MD USA; 5 Clinical Research Center for Brain Sciences, Herzog Medical Center, Jerusalem, Israel; 6 Chairman of Geriatric Division Herzog Medical Center, Jerusalem, Israel

**Keywords:** Pressure ulcer (PU), Chronic diseases comorbidities, Older patients, pathogenesis

## Abstract

**Background:**

The prevalence of pressure ulcers particularly in the frail older adult population continues to be high and very costly especially in those suffering from chronic diseases and has brought a higher awareness to comprehensive, preventive and therapeutic measures for treatment of pressure ulcers. Internal risk factors highlighted by comorbidities play a crucial role in the pathogenesis of pressure ulcers.

**Main body:**

Focusing on the impact of common chronic diseases (comorbidities) in aging on pressure ulcers (e.g., cardiovascular diseases, diabetes, chronic pulmonary diseases, renal diseases and neurodegenerative disorders) and the significant complicating conditions e.g., anemia, infectious diseases, malnutrition, hospitalization, incontinence and polypharmacy, frailty and disability becomes important in developing a more complete, inclusive and multidisciplinary approach to prevention of PU in older patients.

**Objective:**

To describe chronic and acute conditions which are risk factors in elderly patients for developing PU.

**Methods:**

We present an overview of comorbidities seen with PU in three diverse patient locations.

The inclusion criteria are sites (community, acute hospital and long term facilities), older patients, chronic diseases and pressure ulcers grade 2 and over.

Using a recently developed conceptual framework accepted by European and National Pressure Ulcer Advisory Panels, we examined chronic diseases to identify the risk factors of chronic conditions and complicating conditions which potentially influence risk for PU development.

**Conclusion:**

Multiple chronic diseases and complicating factors which associated with immobility, tissue ischemia, and undernutrition are caused to PU in community settings, hospitals, and nursing facilities.

## Background

Life expectancy of older adultshas significantly increased due to better living conditions and improved medical treatment. In Western countries, the average life expectancy increased for women from 70 years to 82 and for men to 80 years [1]. In contrast with the potential to live longer with good quality of life, many older patients in practice suffer from multiple diseases, frailty, and disability, resulting in hospitalizations and transfer to nursing home (NH) residencies [2]. The older patient exhibits declined physiological reserve, and often developschronic diseases, sometimes resulting in frailty, disability and geriatric syndromes [3]. Immobilization, lying in bed for extended periods of time, contributes to the development and appearance of pressure ulcers (PU) [4]. Chronic diseases with prolonged duration stretch patients to the limit of functional reserve. Prolonged chronic diseases can contribute to reduced mobility and weight loss, leading to increased susceptibility for PU.

Sarcopenia and frailty occur as biological changes at the cellular level as well as physiological changes characterized by reduced muscle strength, weight loss, and physical inactivity. Frailty often results in dependency, polypharmacy, disability, geriatric syndromes, institutionalization and early death [5].

Multiple factors act together synergistically to cause PU in the functionally impaired frail older population. The pathogenesis of PU is a multifactorial process involving inflammatory factors, hormonal changes, reduced immune protection, impaired blood perfusion and degenerative changes [6]. A comprehensive approach is required for functionally impaired older patients with co-morbidities to prevent development of PU [7].

Disability and geriatric syndromes are various clinical and functional conditions frequently occurring in older persons affecting activities of daily living (eating, dressing, transfer, walking etc) and quality of life (suffering, pain, loneliness etc) [8].

Some PUs in older adults may be unavoidable, meaning that PU will occur despite optimal care and monitoring. Unavoidable PUs will occur particularly in patients suffering from chronic diseases with an extended duration, stretching a patient’s deterioration to the limit of functional organ failure (heart, kidney and lung). A consensus of NPUAP multi-disciplinary experts in 2010 considered certain situations such as hemodynamic instability (low cardiac output, hypotension) causing inadequate peripheral perfusion and severe undernutrition (including the refusal of artificial nutrition) to be triggers for unavoidable ulcers [9].

Pressure ulcers in these patients are the final end of the pathway of accumulating comorbidities of high severity with complicating conditions affecting some frail or disabled elderly patients.

The prevalence of PU particularly in older adults continues to be disturbingly high especially in those suffering from chronic diseases and frailty, bringing a higher awareness to applied preventive and therapeutic measures [10]. An overall pressure ulcers prevalence of 18.1% highlighted a 2007 European pilot survey undertaken by the European Pressure Ulcer Advisory Panel (EPUAP), which included 5947 hospital patients located in several countries [11]. A survey conducted by the International Pressure Ulcer Prevalence Survey found hospital pressure ulcer prevalence varied from 8 to 14% in hospital units and incidence varied from 3 to 5% [12].

Coleman et al. 2014 [13]. recently developed a conceptual framework of risk factors for PU development. This model is supported by the major PU professional advisory committees: NPUAP and EPUAP. The framework is based on characteristics classified as key direct, indirect and other potential indirect causal risk factors. Direct causal factors include immobility, skin/pressure ulcer status and poor perfusion. Indirect factors are moisture, sensory perception, diabetes, low albumin and poor nutrition. Other potential indirect factors include old age, medications, pitting edema and other factors relating to general health status including infection, acute illness, raised body temperature and chronic wounds [13]. Based on Coleman’s model, we examined various chronic diseases frequently seen in older adults in order to elucidate further the intrinsic risks for PU.

The etiological mechanisms by which stress and internal strain interact with damaged skin and subcutaneous tissue resulting in pressure ulcer development include localized ischaemia, reperfusion injury, impaired lymphatic drainage and sustained cell deformation [14].

Pressure ulcers are typically accompanied by severe complications including pain, depression and infections. These complications induce further health deterioration, with prolonged patient suffering, longer hospitalization, early mortality and increased cost [15]. Pressure ulcers are a heavy burden on the health care team, especially the nursing staff, and require increased care by the medical personnel in all settings. As a result, there is a substantial increase in overall cost [16]. In a systematic review of the health conditions in hospital and nursing homes (NH) residents, PU are cited as one of the seven causes associated with short term mortality [17].

## Main body

### Comorbidities and pressure ulcers development

Identifying the impact of comorbidities is of critical importance in understanding the development of pressure ulcers. Comorbidities can be defined as concurrence of multiple chronic diseases in the same patient. The population with comorbidities differs from those with individual chronic diseases due to the interaction between the multiple conditions, leading to the need for a comprehensive and multidisciplinary approach and long term continuing care. Chronic medical conditions are significantly more prevalent among older adults. Eighty-one percent of Americans over sixty-five have more than one chronic condition Many of these people have five or more chronic conditions, associated with higher rates of ED visits and hospitalizations as well as more prescriptions [18]. Older adults with multiple chronic conditions are also more likely to have physical disabilities and social isolation. The occurrence and prevalence of PU with comorbidities varies according to the setting. Each setting has a different rate of advanced chronic conditions among older adults and as a result, a different prevalence of PU specific to that setting. In community settings there is a lower rate of PU occurrence due to less immobility and malnutrition from advanced chronic illness. In the outpatient setting the prevalence of pressure ulcers was found to be 1.61% of 75,168 older individuals and increased to 4.2% for those over the age of 85 with adjusted RR of 5.06 [19]. Older patients with serious acute conditions are treated primarily in a hospital setting with a notable increase in PU development within a short length of stay. In the hospital setting, the PU incidence rate in one study was found to be 4.5% and the prevalence rate on admission was 5.2% [20]. Long term care patients have the highest rate of PU development associated with higher frequency of mortality and advanced severe chronic conditions. The prevalence of PU in two long-term care facilities in Canada was found to be 36.8 and 53.2%, respectively. The incidence rate was 11.7 and 11.6% [21]. The Skilled Nursing Department (SND) in geriatric hospitals typifies long term care settings in accepting bed bound patients and those affected with one or more of the following complications with a background of multiple comorbidities: extensive and deep PU (grade 3–4); cancer requiring palliative care; renal failure dependent on haemodialysis; continuous oxygen; non-invasive ventilation; and tracheostomy. In a SND study 61.5% of the patients exhibited PU at admission [22].

The common age-related chronic diseases are identified as cardio-vascular, diabetes, lung, renal, musculoskeletal, neurodegenerative diseases. Exploring the impact of these diseases provides insight into multidimensional effects on older patients regarding clinical symptoms, complications, consequences and effectiveness of treatment. Progression of these diseases can be manifested through impaired motor, sensory, immune and hormonal systems and lead to frailty, disability, geriatric syndromes and isolation. The combined effect of these impaired systems and organs may result in associated complicating conditions including; malnutrition, anemia of chronic disease, recurrent infection, polypharmacy and hospitalization. The significance of comorbidity risk factors in the pathogenesis of PU requires further investigation, recognizing the insolvability of PU prevention solely with external relief devices.

## Methods

The inclusion criteria for the literature review are studies of PU risk factors in the community, in acute hospitals and in long term facilities, A medline search was done of PU risk factors and older adults and community; and PU risk factors, older adults and hospital; and PU risk factors, older adults and long term care. We further searched under PU and “chronic conditions” and “pressure ulcer risk factors” with specific common chronic diseases: diabetes, congestive heart failure, cardiovascular disease, stroke, peripheral vascular disease, chronic kidney disease, chronic pulmonary disease/COPD, dementia/neurodegenerative disease, osteoarthritis, osteoporosis, obesity. After reviewing this literature, we did a search of pressure ulcer risk factors and specific associated problems: incontinence, anemia, malnutrition, polypharmacy. We excluded studies looking only at stage 1 ulcers or medical-device related ulcers and excluded studies including only spinal cord injury patients.

## Results

### Cardiovascular diseases

Advanced heart disease with low cardiac output and/or decreased oxygenation, results in hypotension, decreased blood perfusion and peripheral ischemia contributing to the appearance of PU [23].

Atherosclerosis is the primary physiological process affecting the vascular system in older adults. Well-known risk factors for atherosclerosis include smoking, high blood pressure, hyperlipedemia, diabetes and sedentary lifestyle. The accelerated atherosclerosis process decreases blood perfusion to essential organ targets such as the heart, brain, eyes, kidneys, legs and soft tissue skin predisposed to local ischemia. Reduced perfusion to skin and soft tissue may contribute toPU development. Cardiovascular disease is an often cited risk factor for PU among ICU hospital patients [24]. Among hospital patients after myocardial infarction, reduced LV ejection fraction predicts PU [25]. CHF was also found to be a risk factor for PU among post-surgical ICU hospital patients [26].

In our review, we did not find consistent associations between hypertension and PU.

Peripheral vascular disease (PVD) is distinguished by arterial, venous and/or lymphatic insufficiencies, particularly prominent with the onset of PU. Heel ulcers are associated with peripheral arterial disease among hospitalized patients [27]. Another study of hospital patients with vascular disease found that low ABI was a risk factor for all PU [28].

Direct causal risk factors from Coleman’s theoretical model associating PU and vascular diseases are primarily poor perfusion due to local ischemia, delayed reperfusion of ischemic tissue and impaired lymphatic drainage, all reducing tissue tolerance and the threshold to develop PU. Indirect causal factors might be diabetes and poor nutrition. Other potential indirect factors in the Coleman model might include side-effects of cardiac, diuretic and cholesterol medication (causing hypotension and muscle pain), old age and other factors relating to general health status including acute illness. Edema or dehydration as a result of congestive heart failure (CHF), could cause structural changes of the skin layers, promoting the development of PU.

In patients with advanced vascular disease, PU prevention might include stroke prevention and edema management as well as good nutrition and muscle strengthening.

Patients with cerebrovascular accidents (CVA) are prone to be immobilized and to develop PU. Typically, stroke results from atherosclerotic cerebrovascular disease. Therefore, poor perfusion and medication side effects could also contribute to PU after stroke, particularly during hospitalizations. Stroke also increases risk for falls and injuries leading to more hospitalizations and disability. Post-stroke infection such as pneumonia is also common and may predispose to PU.

In a study of stroke patients in Thailand living in the community, PU prevalence was very high and was associated with malnutrition, friction, and moisture [29]. A study in the UK found that one year after stroke, PU prevalence was high (above 20%) both among people living at home and among people living in the community [30].

In an outpatient setting, Margolis et al. [19] found that the prevalence of CVA among patients with PU was 13.1% with an adjusted RR for PU of 1.57. The prevalence of CHF was 13.4% and the adjusted RR for PU is 1.39; Deep vein thrombosis (DVT) prevalence was 2.2% with adjusted RR for PU 1.39. Lower limb edema prevalence was 17.2% with adjusted RR for PU 1.37.

In a retrospective study of over 50,000 patients, Lyder et al. [20] found that of the 2313 patients with hospital acquired pressure ulcers 33.9% suffered from CVA compared to the patients without pressure ulcers of whom only 22.4% suffered from CVA (*p* < .001). 1013 (43.8%) patients with pressure ulcers suffered from CHFcompared to the patients without pressure ulcers of whom only 28.4% suffered from CHF (*p* < .001).

In patients after stroke, PU prevention might include usual rehabilitation therapy, occupational therapy assessment, secondary stroke prevention, fall prevention screening and assessment, and assessment and management of continence.

### Diabetes mellitus

The prevalence of diabetes in the last several decades has increased [31] and is expected to increase much more as populations age and become more obese.. Lack of sensory perception from diabetic neuropathy is a major risk factor to non-healing wounds, especially diabetic foot ulcers and PU. Diabetic neuropathy also causes diabetic foot syndrome and Charcot foot, leading to bone destruction, deformity and infection, all increase to formation of diabetics ulcers [32]. Distinguishing between PU, diabetic and ischemic ulcers can be difficult and there are overlap signs [33]. Like pressure ulcers, ischemic ulcers arise from to pressure on bony prominences. Diabetic ulcers typically are well-demarcated with hyperkeratotic margins. In ischemic ulcers, the foot is typically cool with notable skin atrophy. Many years of diabetes contributes to glycosylated vessels impairing perfusion resulting in local ischemia to the skin. Many diabetic patients suffer from vascular complications in various target organs, accelerating the atherosclerotic process resulting in local ischemia [34]. Hypoglycemic medications may cause decreased activity from hypoglycaemia and weight gain. Diabetes also increases risk for infections and the importance of infection is described below.

In studies of risk factors for PU, diabetes is the chronic disease most commonly identified in any care setting. Margolis et al. [19] reported that the prevalence of diabetes was 7.4% in older adults in the community and the adjusted RR for PU with diabetes was 1.75. Lyder et al. [20] found that of 2313 patients with hospital acquired pressure ulcers, 971 (42.0%) suffered from diabetes. This was significant (*p* < .001) compared to the patients without pressure ulcers of whom only 33.4% suffered from diabetes. In populations of hospitalized hip fracture patients [35] and critically ill hospital patients [24], diabetes was a risk factor for PU. In a related meta-analysis, diabetes doubles the risk of post-operative PU [36]. In a study of 59 nursing facilities in 8 European countires, diabetic residents had higher rates of PU than non-diabetic residents [37]. Heel ulcers are particularly prevalent among diabetic patients [27]. Not surprisingly, heel ulcers tend to be associated with leg ischemia. Also, diabetic feet have stiffer and sometimes thinner plantar skin. This increased heel skin stiffness may contribute to heel ulcers [38]. Diabetes results in drier [39], stiffer, thinner skin [40] which may increase risk for PU beyond the feet.

Furthermore, diabetes is associated with obesity. Morbid obesity is significantly associated with PU among hospital patients [41] and nursing home patients [42].

In older adults with prolonged diabetes and microvascular or macrovascular complications, PU prevention might include good nutrition, lower extremity muscle strengthening, minimizing bedrest when ill, appropriate glycemic management but avoidance of hypoglycemia, stroke prevention, use of skin moisturizers, and avoidance of conditions which dry the skin (very hot baths, low humidity). In some cases, PU wil be unavoidable in immobilized diabetic patients.

### Chronic pulmonary disease (CPD)

Pulmonary function declines with age. Vital capacity decreases, airway dead space increases, and the ventilatory response to hypoxia declines. Pulmonary disease, obesity and smoking habits further reduce lung function. An association between advanced chronic pulmonary disease and PU is partially explained by decreased oxygenation causing tissue hypoxia. Inflammation, increased work of breathing, oral glucocorticoids, loss of appetite, and decreased physical activity from chronic pulmonary disease all contribute to muscle wasting. In the Coleman model, direct causal risk factors associating PU and advanced chronic pulmonary disease could include immobility and poor perfusion. Indirect factors could include sarcopenia. Other potential indirect factors from the Coleman model could include old age, medications (such as steroids which increase edema and affect the skin structure), infections and hospitalizations. Also cigarette smoking, which likely caused the chronic lung disease, may have caused generalized atherosclerosis reducing skin perfusion. The use of medical devices including cords from oxygen supplying respirators, tracheostomy cannula, and tubing cause compression of facial and neck skin tissue, predisposing to the development of PU [43]. The relative risk (RR) of pressure ulcers among patients with CPD in a rehabilitation unit was found to be 1.92 [44]. Margolis et al. [19] reported that the prevalence of CPD was 9.0% in older adults in the community and the adjusted RR for PU was 1.24. Among older adults receiving home health services, home oxygen use is associated with PU incidence [45]. Lyder et al. [20] found that of 2313 patients with hospital acquired pressure ulcers, 810 (35.0%) suffered from CPD. This was significant (*p* < .001) compared to the patients without pressure ulcers of whom only 28.4% suffered from CPD In a European study of hospitalized patients after a hip fracture, pulmonary disease was a risk factor for PU [46]. Similarly, pulmonary disease has been associated with heel PU among hospitalized orthopaedic patients [47].

In order to prevent PU, older adults with severe pulmonary disease should optimize nutrition, manage edema, minimize bedrest when ill, and optimize activity and endurance.

### Kidney disease

Kidney functioning decreases with the aging process, with substantial but varying declines in glomerular filtration rate and renal blood flow. Common age-related diseases such as diabetes and hypertension further impair kidney function. An association between chronic kidney disease and PU is explained due to imbalance of homeostasis functions (body fluids, electrolytes disturbance and hormonal losses) reflected in impaired concentration and dilution, anemia, and soft tissue changes. In the Coleman model, direct causal risk factors associating PU and chronic kidney disease could include poor perfusion and pre-existing skin/pressure ulcer status. Indirect causal factors could include low albumin and poor nutrition. Other potential factors include old age, medication (antihypertensive medication could lower blood pressure, reducing perfusion and reducing activity while steroid affect the skin as noted above), infection, and acute illness. Among hospitalized surgical ICU patients in one study, a diagnosis of renal disease was associated with PU (adjusted OR 1.75) [26]. Although some of the association between end stage renal disease and PU is likely due to underlying conditions, chronic kidney disease [48] and hemodialysis [49] are associated with xerodermia [50] and with impaired wound healing.

PU prevention for older adults with chronic kidney disease can include good nutrition, management of edema, prevention of stroke/MI, and avoidance of hypotension.

### Musculoskeletal disorders

Our literature search did not find consistent significant associations between osteoarthritis and PU or osteoporosis and PU. However, hip fracture, which is typically due to osteoporosis is associated with high rates of PU, particularly in the hospital setting [51].

### Neurodegenerative disorders

Neurodegenerative disorders including dementia, Alzheimer’s disease and Parkinson’s disease, are common with advanced age. With increased longevity, prevalence rates are increasing. According to current statistics, dementia is an increasingcause of mortality [52]. Although dementia is typically viewed as a neurodegenerative disease without systemic implications’ Gibson et al. [53] suggested that Alzheimer Disease (AD) can be viewed to include systemic effects possibly related to oxidative stress from mitochondrial dysfunction. These systemic effects of Alzheimer disease can result in motor, sensory, autonomic, cognitive, or behavioral changes predisposing to PU [54].

The tendency towards being bedridden, immobile, and exhibiting flexion with severe spasticity seen in advanced dementia and the rigidity in Parkinson disease, contribute to the increased propensity towards pressure ulcers. Mitchell et al. [55] describes the course of advanced dementia including difficulties with eating and swallowing, lack of proprioception and sensation, episodes of fever and infections and increased agitation related with the development of PU. Poor nutrition due to cognitive impairment and social isolation may also contribute to the association with PU, as well as problems with bathing and toileting in late stage dementia. Almost 40 % of advanced dementia patients developed pressure ulcers before death [55]. There is reason for concern that increasing rates of advanced dementia will lead to higher rates of PU, resulting in increased suffering and mortality [56].

Direct causal risk factors, according to the Coleman model, associating PU and Neurodegenerative disorders would primarily be immobility. Indirect factors could include poor nutrition. Other potential indirect factors could include old age, sedating medication causing decreased activity and oral intake, infection, falls with injuries and chronic wounds.

Recent studies indicate patients with advanced dementia have a significantly higher prevalence of PU, compared to other comorbidities [56]. Among hospitalized patients with hip fracture, dementia or delirium is associated with incidence of PU [35]. A SND research study demonstrated that advanced dementia is highly significantly associated with PU in multiple regression analyses (OR = 3.0, 95% CI: 1.4–6.3; *P* = 0.002) [22]. In a study of American nursing facilities, dementia was a predictor of PU incidence despite consistently good care [57]. PU is particularly common in patients with dementia near the end of life. Some studies find that older adults with dementia are less likely to have PU, possibly because in early dementia, many people will be physically robust. Margolis et al. [19] reported that the prevalence of Alzheimer’s disease was 3.9% among older adults in the community and the adjusted RR for PU was 2.01; while Parkinson’s disease prevalence was 2.2% among older adults in the community and the adjusted RR for PU was 2.34. In patients with advanced dementia, early intervention by monitoring caloric and protein intake might slow the development of comorbidities including the development of PU. PU prevention in advanced neurodegenerative disease might also include management of continence (with supportive care), regular physical activity (even including restorative care for bedbound patients), minimizing bedrest in the setting of acute illness, fall prevention, and avoidance of sedating medications.

## Complicating conditions

Many people with advanced chronic conditions will develop complications which contribute to the development of PU.

For example, common conditions which complicate chronic illness and occur in all medical settings include; malnutrition, anemia of chronic disease, recurrent infectious diseases, hospitalization and polypharmacy.

### Malnutrition

Due to many of the above chronic conditions, many patients will experience dysphagia or anorexia and as a result lose weight.

Low intake of calories and protein causes sarcopenia and results in decreased strength of the lower extremities, frailty, and risks for falls with injuries, hospitalizations, and immobilization. Malnutrition also impairs immune and hormonal function, causes skin changes (epidermis, dermis), reduces subcutaneous tissue and causes muscle atrophy, all increasing vulnerability to PU [58].

In a cross-sectional study of hospitalized and nursing home patients, Shahin et al. [59] found a significant relationship between malnutrition parameters such as weight loss, BMI < 18.5, poor nutritional intake and PU. Low BMI is highly associated with PU due to inadequate nutrition and reduced tissue thickness of the skin and subcutaneous tissues [60]. In studies of hospitalized American ICU patients (Hyun, AJCC, 2014) and hospitalized patients in Australia, the incidence of PU was highest among those who are underweight or extremely obese (Hyun, AJCC, 2014).

Pinchcofsky and Kaminski [61] reported 65% of the severely malnourished residents in LTC settings had PU in contrast to none in those with even mild to moderate malnourishment. Margolis et al. [19] reported that the prevalence of malnutrition was 0.4% among older adults in the community and the adjusted RR for PU was 3.06. In a hospital based setting, the patients suffering from malnutrition were twice as likely to develop PU (RR = 2.1; 95% CI 1.1–4.2) compared to patients with adequate nutrition [20]. Similarly, in a Skilled Nursing Department setting comparing patients with PU and without PU, a significant association was found by univariate analysis: low weight and low BMI (< 22) were found in the PU group; 61 kg vs 68 kg, *p* = 0.02; BMI 22.9 vs 25.5 *p* = 0.007 and in multivariate analysis: BMI was 56% versus 44% OR: 0.92 95% CI: 0.86–0.99 *P* = 0.02 [22].

Despite the significant association between malnutrition and PU, recent systematic and randomized control trials have found that improving nutrition is only modestly effective in the prevention and treatment of PU [58]. In a meta-analysis of eight trials of 6062 participants comparing the effects of mixed nutritional supplements with standard hospital diet, no clear evidence emerged of an effect of alimentary supplementation on pressure ulcer healing (pooled RR 0.86; 95% CI 0.73–1.00; *P* value 0.05) [62]. The pro-inflammatory cachexia state (due to advanced chronic illness) may contribute directly to PU development, beyond simply causing weight loss and undernutrition. Similarly, hypoalbuminemia is a powerful predictor of PU as well as mortality and other poor outcomes. Although hypoalbuminemia is popularly perceived to be caused by malnutrition, acute and chronic illnesses often cause hypoalbuminemia, and acute illness may be the primary driver of the association [63, 64].

As Thomas noted, in these studies, undernourished patients are often hospitalized for a longer stay in intensive care units, with a reduced functional status, and an increased acuity of illness, higher comorbidities and higher mortality [65]. The challenge for the geriatrician, medical team, and supporting health staff is to distinguish patients with severe chronic illness who might benefit from the potential prevention and treatment of PU with nutritional support in addition to management of chronic conditions. Those patients with severe, long term chronic illness continue to require nutritional monitoring, although this by itself is not likely to reverse the PU. Strategies for improving body weight must include optimizing alertness and managing infection and inflammation. In many cases, PU will be unavoidable in severely undernourished/underweight older adults despite consistently good care.

### Anemia

Anemia is a condition in which the body lacks sufficient red blood cells supplying oxygen to body tissues. The changes in oxygen dissociation-curve seen with anemia affect the risk for tissue ischemia and may contribute to PU development.

Anemia of chronic disease seen with chronic renal failure, inflammatory disease and cancer reflects advanced chronic illness, predisposes to frailty and adverse outcomes and influences the process of healing.

A study comparing 174 patients with and without PU, suffering from multiple comorbidities, and hospitalized in the Skilled Nursing Department for three and half years, found that anemia of chronic disease is significantly associated with PU both in univariate analysis (51.4% vs 32.8%, *p* = 0.01 OR:2.16, 95% CI:1.14–4.08) and multivariate analysis (63% vs. 37% *p* = 0.004 OR:0.73, 95% CI: 0.58–0.90) [22]. In a study of long-term care residents with PU, anemia was associated with non-healing over six months [66]. In the hospital, one study found that low admission hemoglobin is associated with higher incidence of PU after hip fracture [35]. Margolis also studied anemia as a risk factor for PU among elderly in community dwellings but found the adjusted RR to be 1.03 reflecting a minimal relationship [19]. However, in a study of community-based older adults receiving home health care, anemia predicted PU incidence [67].

In a specialized retrospective study, Keast [68] demonstrated that the administration of erythropoietin to four anemia patients with very deep and severe PU grade IV wounds, improved the mean haemoglobin level and was associated with a decrease in the wound surface size and depth.

Poor perfusion is the direct causal risk factor in the Coleman model associating PU and anemia. Indirect causal factors might be poor nutrition. Other potential risk factors might include old age and reduced activity.

The decreased hemoglobin associated with chronic diseases can occur due to the effect of inflammatory cytokines on erythroid progenitor cells. Erythropoitin can modulate inflammation and stimulate wound healing [68]. Hepcidin is a peptide hormone secreted by the liver during inflammation. It induces trapping of macrophages and liver cells, and decreases availability of iron for erythropoeisis resulting in anaemia of chronic disease [69]. Blood transfusion might be an important tool in the treatment of PU in patients with low haemoglobin. Erythropoietin and intravenous iron supplementation (if there is concomitant iron deficiency) and other supplements (if there is concomitant vitamin B12 or folate deficiencies) are used in PU patients with anaemia of chronic disease.

### Infectious diseases

Infection is invasion of bacterial pathogens causing a catabolic state resulting in breakdown of tissues. The health deterioration with concurrent infections delays and even prevents wound healing, thus significantly increasing risk to development of PU. Increased skin temperature also is an indirect causal risk factor for PU.

Malnutrition, dysphagia, and urinary catheters, which are common problems in patients with advanced chronic disease, are risks for infection including aspiration pneumonia, urinary infections, as well as infections connected with PU including soft tissue, sepsis and osteomyelitis. Quick treatment of infection (and certainly prevention) may reduce the propensity for developing skin damage and PU.

Margolis et al. [19] reported that the prevalence of urinary tract infection and pneumonia was 18.2 and 21.1% among older adults in the community and the adjusted RR for PU was 1.19 and 0.79 respectively. In the SND study comparing patients with and without PU, the use of antibiotics was significantly higher in the group with PU and higher infections, 50.5% versus 38.8%; *p* = 0.001 with OR: 2.26, 95% CI: 1.22–4.20^21]^. Khor [70] reported that hospitalized patients with pressure ulcers who had infected grade IV wounds or high neutrophil count (presumably due to infection) displayedearly mortality (infected deep pressure ulcers HR = 2.21, *p* = 0.006 and neutrophilia HR = 1.76; *p* = 0.031) compared to other hospital patients with PU.

In a study of palliative care patients across multiple settings, infection was associated with PU [71].

Infection prevention strategies for vulnerable older adults include appropriate vaccinations, hand washing, avoidance of indwelling catheters. There are specific guidelines for preventing post-operative infections and ventilator associated pneumonia.

### Incontinence

Many of the chronic conditions discussed here are associated with urinary and/or fecal incontinence, either due to advanced disease or due to side effects of medications.

In a study in a California hospital, 73% of patients with PU had fecal or urinary incontinence [72]. In a review of PU among nursing facility residents, fecal incontinence was associated with PU incidence [73]. Among 3000 patients admitted to home care, incontinence was associated with prevalence of PU [74]. In another home care study, fecal incontinence was associated with incidence of new PU [75]. In biomedical engineering simulation, compression and shear with wetness in aged skin produced the highest skin surface loads [76]. Moisture can cause direct chemical damage to skin and can act indirectly, reducing the load needed to cause tissue damage.

For high risk older adults, continence screening and assessment can be helpful.

### Polypharmacy

Older adults who take at least 5 medications are likely to be experiencing adverse effects from their medications due to drug-drug interactions and drug-disease interactions. Several types of medications are associated with PUs including sedatives [77], vasopressors [78], and corticosteroids [20]. Sedatives and corticosteroids in particular are often prescribed to older adults with advanced chronic illness. Sedatives increase immobility and may reduce oral intake. Presumably those effects would also be seen with anticholinergic medications or other drugs which have the adverse effect of sedation. Vasopressors may reflect hemodynamic instability and critical illness or may be contributing to skin ischemia. Corticosteroids may contribute to skin atrophy and impaired wound healing.

### Hospitalization

Many pressure ulcers develop during acute hospitalizations. Incidence rates vary among studies and there seems to be regional variability. Hospitalized patients spend more time in bed than they would in the community. Moreover, they often receive new medications which may be sedating or reduce appetite. One-third of hospitalized older female patients experience functional impairment from their hospital stay and only half will recover to their pre-hospital function [79]. Acutely ill older patients treated in a “hospital at home” program display better nutritional status [80] after their acute illness and a lower likelihood of nursing home placement [81] than patients treated in a hospital. Avoiding hospitalizations through intensive ambulatory care and home care may be able to prevent functional decline and nutritional decline that lead to PU.

## Discussion

The main objective of this article was to detail the chronic and acute disease risk factors for frail elderly patients developing PU.

Multiple chronic conditions impair mobility, and also have a cumulative vascular, inflammatory, immune, hormonal and degenerative effect on the development and treatment of pressure ulcers. Different locations of pressure ulcers (sacrum v. heel) may have different disease associations. For example, heel ulcers seem more strongly associated with diabetes and PVD than sacral ulcers.

Identifying the key risk factors and impact of comorbidities and associated geriatric conditions on the susceptibility of the elderly patient is of critical importance for the prevention of pressure ulcers.

The authors of this overview used the Coleman conceptual framework focusing on pathways for risk factors in PU development identified with comorbidities. The Coleman conceptual model does not list chronic conditions other than diabetes as causal factors for PU. However, many of the chronic conditions discussed here fit into the Coleman model. These are particularly important as rates of diabetes, dementia, and severe obesity are expected to increase dramatically in the coming decades.

**Fig. 1:**
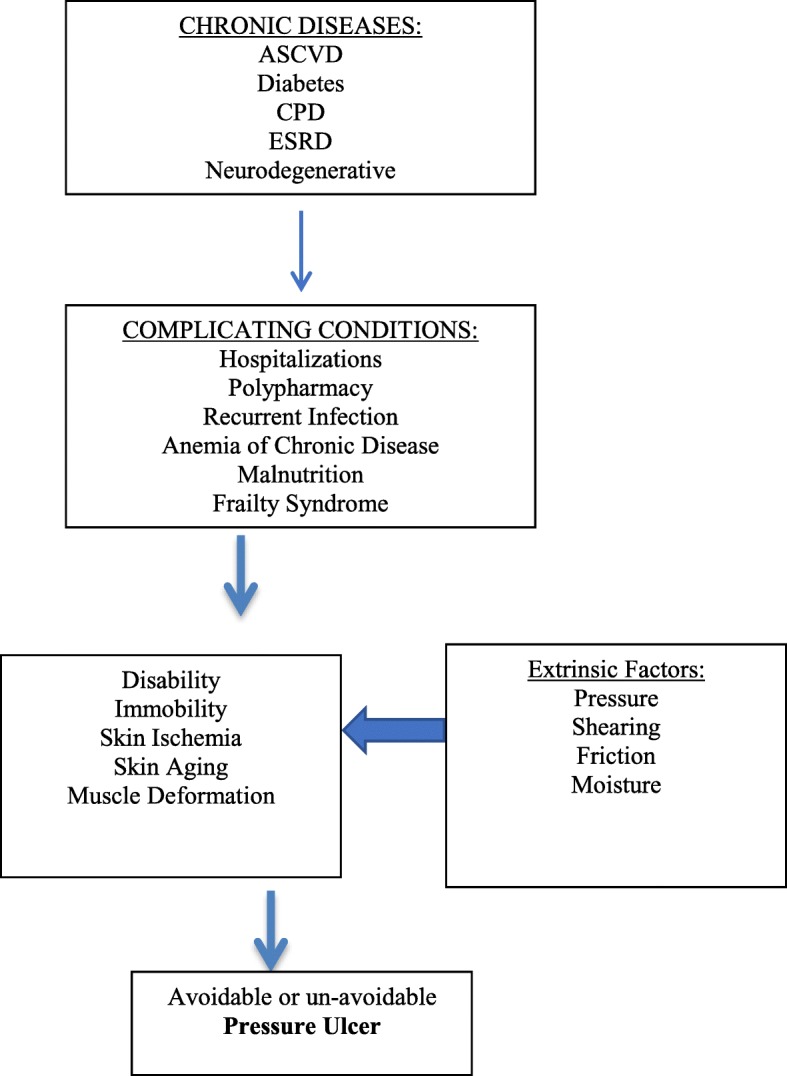
Conceptual Model of Development of Pressure Ulcers in Older Adults

Direct PU causal factors associated with comorbidities include immobility, skin/pressure ulcer status and poor perfusion. Indirect factors are moisture, sensory perception, diabetes, low albumin and poor nutrition. Other potential factors include old age, medication, pitting edema and other factors relating to general health status including infection, acute illness, raised body temperature and chronic wounds (See Fig. 1).

The direct risk factor associating PU and cardiovascular diseases is primarily poor perfusion occurring in all medical settings. Diabetes directly influences PU by risk factors of poor perfusion and skin changes and indirectly by lack of sensation perception in all medical settings. Direct risk factors associating PU and Chronic Pulmonary Disease (CLD) include immobility and poor perfusion. The primary direct causal factor associating PU and neurodegenerative disorders is likely immobility and the indirect causal factor of poor nutrition in both community and LTC settings. Effects seem strongest for diabetes, stroke, and advanced dementia. For heel ulcers, peripheral vascular disease and diabetes are particularly strong causal factors. PU may be unavoidable in patients with these conditions, given circumstances such as reduced mobility and undernutrition.

## Conclusion

Among chronic diseases, diabetes, stroke, and advanced dementia seem to be most strongly associated with PU development. Immobility patients with low BMI, low albumin and haemoglobin, high inflammatory markers (CRP and ESR)associated with these conditions and with hemodynamic instability (low cardiac output, hypotension) and chronic complications may be most likely to lead to unavoidable PU (9).

The population with comorbidities differs from those with individual chronic diseases due to the interaction between the multiple conditions, leading to the need for a comprehensive and multidisciplinary approach and chronic continuing care. Complicating conditions are identified with the progression of diseases to later advanced stages where the modifying impact of comorbidities on the aging process becomes strongly prevalent in contributing to development of PU.

It is important to understand the PU risk factors coming directly from multiple chronic diseases, complicating factors, functional impairment and disability and the unavoidable PU (Fig. 1). Understanding the pathway to immobility, tissue ischemia and undernutrition to develop PU is crucial. As a geriatrician that looks at the whole patient and s the clinical course of the patient, managing chronic diseases and their complications is the core of prevention and treatment for avoidable or unavoidable PU.

Appreciating PU as a dreaded complication from advanced chronic comorbidities and associated health conditions can help guide treatment goals. Thus the patient, family, and health care team are empowered to improve prevention and optimize treatment of the wounds (managing anemia, optimizing oxygen and blood supply, maintaining mobility and muscle strength, minimizing bedrest, stroke prevention, judicious use of antibiotics and careful attention to medication side-effects as well as optimizing nutrition (and careful weight monitoring) together with more traditional interventions such as pressure relief devices and repositioning serving as the optimal treatment for pressure ulcers), and to change the treatment priorities to control the symptoms of PU (unavoidable PU) and ultimately enhance the quality of life for the elderly patient.

## Abbreviation

PU: Pressure ulcers
